# Risk aversion in risk‐taking tasks: Combined effects of feedback attributes and cognitive reflection ability

**DOI:** 10.1002/brb3.2957

**Published:** 2023-03-22

**Authors:** Wei Li, Siliu Chen, Zhibing Xiao, Dandan Li, Chenyu Lv, Shuyue Zhang, Ofir Turel, Qinghua He

**Affiliations:** ^1^ Faculty of Psychology and MOE Key Laboratory of Cognition and Personality Southwest University Chongqing China; ^2^ Department of Psychology, Faculty of Education Guangxi Normal University Guilin China; ^3^ Computing Information Systems The University of Melbourne Parkville Victoria Australia; ^4^ Southwest University Branch Collaborative Innovation Center of Assessment toward Basic Education Quality at Beijing Normal University Chongqing China; ^5^ State Key Laboratory of Cognitive Neuroscience and Learning Beijing Normal University Beijing China

**Keywords:** cognitive reflection ability, curiosity theories, feedback effects, risk‐taking

## Abstract

**Introduction:**

Feedback on human choices is important because it can affect risk‐taking and rationality in subsequent decisions. In daily life, choices are not always followed by immediate outcomes nor are they always followed by simple, single‐dimensional feedback. Here, we seek to extend previous studies on the effects of feedback on subsequent risk‐taking in three experiments.

**Methods:**

We examine whether (1) the effect of feedback immediacy on participants’ risk‐taking exists in tasks containing explicit probabilistic outcome values; (2) increasing feedback dimensionality from one dimension (only about the outcome) to include a second dimension (also about the “rationality” of prior choices) increases feedback effects on risk‐taking; and (3) cognitive reflection ability moderates feedback effects on risk‐taking.

**Results:**

Results showed that feedback reduced risk‐taking in tasks containing explicit probabilistic outcomes (Studies 1 and 2). They further showed that two‐dimensional feedback produces a stronger reduction in risk‐taking compared to single‐dimensional feedback (Study 3). Lastly, results suggested that cognitive reflection ability moderates the effects of feedback on risk‐taking (Study 4).

**Conclusion:**

Taken together, the findings extended the understanding of risk‐taking and mitigating mechanisms and pave the way for intervention studies aimed at changing risky behaviors.

## Graphical abstract

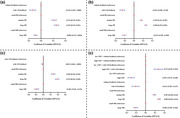

## 1 INTRODUCTION

Choices in life can, but do not always, produce immediate feedback. For instance, when we buy financial products, we often must wait to observe gains or losses, but when we play a slot machine, we typically know the outcome immediately. In addition, the feedback on choices can vary in dimensionality, ranging from feedback on only the outcome (e.g., “you win”) to feedback that also includes information on the decision‐making process (e.g., “you win, and your decision is rational”). For example, on a test paper, students typically not only get a score, but also get feedback on specific errors. Such feedback is important in everyday life because it can be instrumental for further decision‐making (Ilgen & Moore, 1987).

In gambling tasks, under uncertainty, people usually tend to develop strategies. Immediate feedback enables them to re‐evaluate and monitor their strategies; it consequently influences their subsequent choices (Brand, 2008; Ernst, 2005). This has been demonstrated, for instance, in the Balloon Analogue Risk Task (Pleskac, 2008). Regret aversion is an important adjustment mechanism that can contribute to subsequent decision adjustments (Berndsen et al., 2004; Dijk & Zeelenberg, 2002; Zeelenberg et al., 1996; Zeelenberg & Beattie, 1997). Regret is an emotion that stems from reflections on previous actions or inactions that seem like a mistake. It enhances learning, and can consequently influence subsequent decision‐making (Zeelenberg, 1999). Because it is an unpleasant emotion, people try to avoid it (Zeelenberg et al., 1996). The mechanism through which it operates includes not only noticing feedback that suggests that the actual outcome is worse than the expected one, but also realizing that prior choices were suboptimal or incorrect (Zeelenberg, 2002). As such, people are generally inclined to make choices that minimize regret rather than risk (Zeelenberg et al., 1996), and this provides an explanation for why people sometimes prefer the safer options and sometimes prefer the riskier options (Tochkov, 2009). In addition, other studies suggest that expected regret drives people to think more carefully before making decisions, which can make their choices more “rational” (Janis & Mann, 1977).

Feedback mechanisms have been accelerated and have become prevalent with modern technologies. Hence, there is a stronger need to understand how people process and react to the feedback information they are flooded with. Traditional views of rational decision‐making suggest that individuals will evaluate and combine all available information, and that more information should yield better decisions. However, in real life, human decisions cannot be completely rational (Simon, 1955; Tversky & Kahneman, 1974). This is because humans are not always motivated or have the capacity to engage in energy‐consuming reflections; instead, they sometimes act impulsively or take mental shortcuts and avoid full information processing (Kahneman, 2011). Consequently, people often use simple, fast, and frugal heuristics when facing too much information (Todd, 2007). The amount of information we have got does not represent the amount of information that can be used or processed. According to the “more important dimension” hypothesis (Kray & Gonzalez, 1999; Slovic, 1975), decision‐makers will systematically determine their choices by selecting the alternative that was superior on the more important dimension, and imply paying less attention to other dimensions.

Cognitive refection captures the ability or disposition to resist hasty reflections, and to consequently engage in more thorough (Frederick, 2005). Hence, it can be highly relevant for understanding motivation and the ability to process complex feedback and risky decisions. Studies found that cognitive reflection ability is negatively related to risk aversion (Benjamin et al., 2013; Carlos et al., 2016; Donkers et al., 2001; Frederick, 2005; Gill, 2015; Andersson et al., 2016). This is because individuals with high cognitive ability are likely to realize that risk aversion over small stakes is somewhat irrational (Rabin & Thaler, 2001); they have more cognitive capacity compared to others to deliberately evaluate about their choices. This explanation is rooted in dual‐system theory (Evans & Stanovich, 2013; Kahneman & Frederick, 2002; Lilleholt, 2019; Loewenstein & O'Donoghue, 2004), which proposes that humans have two cognitive systems: System 1 and System 2. System 1 processes are fast, effortless, and intuitive, usually associated with heuristic decision‐making, and System 2 processes are slow, effortful, and reflective, typically associated with thoughtful and rational decision‐making (Evans & Stanovich, 2013; Frankish, 2010; Lilleholt, 2019).

There are two characteristics of System 2 related to cognitive reflection: its capacity to monitor System 1's outputs and its capacity to override System 1's functioning (Frederick, 2005). System 2's processes require more working capacity (Evans & Stanovich, 2013), which is highly correlated with cognitive ability (Conway et al., 2003; Süß et al., 2002). Indeed, studies have found that cognitive reflection ability is positively correlated with choices predicted by utility theory in risky choice tasks (Frederick, 2005; Lilleholt, 2019; Oechssler et al., 2009). In addition, it may be worth highlighting that the cognitive reflection test (CRT) provides not only a measure of cognitive reflection ability, but also of impulsiveness. It is possible that participants with high cognitive reflection ability behave more rational because they are less impulsive or because they better understand the decision problems at stake (Lilleholt, 2019).

Synthesizing the above‐mentioned literature, we hypothesize that **(H1)** immediate feedback in tasks containing explicit probabilistic outcome values would reduce risk aversion. This is rooted in the findings that people are generally inclined to make choices that minimize regret rather than risk (Zeelenberg et al., 1996), and prior findings on regret aversion in decision‐making (Larrick & Boles, 1995; Zeelenberg, 1999; Zeelenberg & Beattie, 1997; Zeelenberg & Pieters, 2004). We further hypothesize that **(H2)** this effect will increase when the feedback pertains to more dimensions, that is, people would show less risk aversion in a task with two‐dimensional feedback than in a task with one‐dimensional feedback but would not reach fully rationality. This is based on the regret effect and the “more important dimension” hypothesis (Kray & Gonzalez, 1999; Slovic, 1975). Lastly, we hypothesize that **(H3)** the immediate feedback effect is stronger for people with higher cognitive reflection abilities. This is rooted in dual‐system theories and the role of reflective abilities in motivating and affording information processing (Evans & Stanovich, 2013; Kahneman & Frederick, 2002; Lilleholt, 2019; Loewenstein & O'Donoghue, 2004).

We test these hypotheses in four experiments. In Studies 1 and 2, we aimed to investigate **H1** by comparing choices in a task with immediate feedback (i.e., participants received feedback after each decision) versus a task without immediate feedback, in which participants received aggregated feedback at the end of the task. In Study 3, we tested **H2** by directly comparing the effect of two‐dimensional feedbacks and single‐dimension feedbacks. Lastly, in Study 4, we tested **H3** by examining whether cognitive reflection abilities moderate the effect of feedback. Together, these experiments refine our understanding of feedback attributes and cognitive reflection abilities in the decision under uncertainty situations. The protocol of these studies was reviewed and approved by the local ethical committee and all participants signed the approved informed consent form.

## 2 STUDY 1

The aim of the first study was to examine the effect of outcome feedback immediacy in a task with immediate one‐dimensional feedback (with 1‐D feedback) versus in a task without immediate outcome feedback. Based on regret aversion theories (Larrick & Boles, 1995; Zeelenberg, 1999; Zeelenberg & Beattie, 1997; Zeelenberg & Pieters, 2004), we expected that compared with the task without immediate feedback, people would be less risk‐averse (i.e., more risk‐seeking) in the task with 1‐D feedback.

### 2.1 Method

#### 2.1.1 Participants

Participants included 46 college students (25 females; age: *M* = 19.30 years, *SD* = 1.10, ranging = 18–22). They have a normal or corrected‐to‐normal vision.

#### 2.1.2 Tasks and materials

**FIGURE 1:**
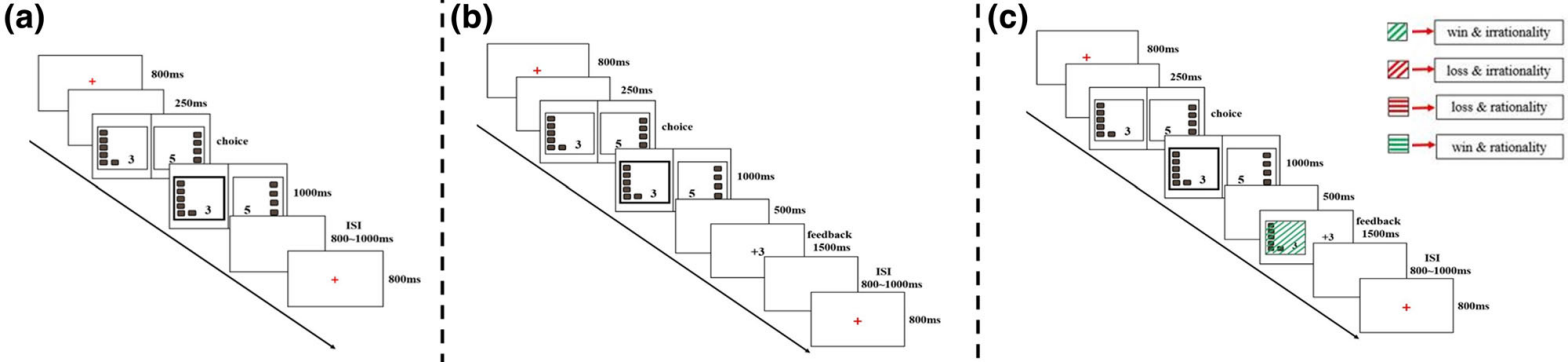
The flowcharts of the task without immediate feedback (a), with 1‐D feedback (b), and with 2‐D feedback (c). In all three tasks, trial started with a red fixation cross for 800 ms. After a short blank screen (250 ms), two options were then displayed side‐by‐side with different magnitude and probability of reward. The reward magnitude was indicated with number of tokens (1–5). The reward probability was illustrated by a stack of 2–8 rectangles, each representing a 10% increment in probability (from 20% to 80%). Here, in this example, participants need to choose between 60% of winning 3 Yuan and 40% of winning 5 Yuan. Participants needed to press the left or the right button corresponding to their choice. Their choice was highlighted (here the left option) with a bold square for 1000 ms after the decision. In the task without immediate feedback (a), no feedback was presented, while in other two tasks, feedback displayed for 1500 ms. The next trial started after a variable ISI (800–1000 ms). In the 1‐D feedback task (b), participants received a message of 1‐D feedback (here, “+ 3” means “win 3 tokens”). While in the 2‐D feedback task (c), the feedback was a 2‐D of either of the four combinations: win & rationality; win & irrationality; loss & rationality; and loss & irrationality (here, win & irrationality).

We used a modified version of the paradigm used in Sharp et al. (2012). In each trial, participants were asked to choose between two options differing in the magnitude and probability of reward (Figure 1). Participants were informed that their goal was to get as many tokens as possible, which were later converted into real money as their payment. We systematically varied the expected values (EVs) of the two options (see Table 1). It was expected that a rational decision‐maker would calculate the EV (consciously or subconsciously), which is the magnitude of reward multiplied by the probability of reward and choose the option with higher EV. To manipulate the various EV combinations of the two options, we calculated the relative EV difference as EV‐ratio (EV1–EV2)/[(EV1+EV2)/2] to relatively balance the attractiveness of each option. We created 14 different combinations, with sizes of EV‐ratio varying from −1 to 1 (see Table 1). Option 1 has a smaller reward magnitude but a larger probability than option 2. According to the EV theory, a positive EV‐ratio indicates that the purely “rational” option is the one with a higher probability of reward (option 1), and a negative EV‐ratio indicates that the purely “rational” choice is the option with higher reward magnitude (option 2).

**TABLE 1:** Combinations of probability and magnitude for each trial

Reward magnitude (coins)		PR	Reward probability (%)		MR	Expected value		EV‐ratio
option 1	option 2		option 1	option 2		EV 1	EV 2	
1	4	3	60	40	20	0.6	1.6	–0.909
1	5	4	70	30	40	0.7	1.5	–0.727
1	4	3	70	30	40	0.7	1.2	–0.526
2	5	3	60	40	20	1.2	2.0	–0.500
1	3	2	70	30	40	0.7	0.9	–0.250
1	5	4	80	20	60	0.8	1.0	–0.222
3	5	2	60	40	20	1.8	2.0	–0.105
3	4	1	60	40	20	1.8	1.6	0.117
4	5	1	60	40	20	2.4	2.0	0.182
1	3	2	80	20	60	0.8	0.6	0.286
2	3	1	70	30	40	1.4	0.9	0.435
3	4	1	70	30	40	2.1	1.2	0.545
1	2	1	80	20	60	0.8	0.4	0.667
2	3	1	80	20	60	1.6	0.6	0.909

Abbreviation: EV, expected value.

***The task without immediate feedback***. On each trial (Figure 1a), a red fixation cross appeared for 800 ms. This allowed participants to get ready. Next, the screen displayed two options, one on the left side and the other on the right side. They had different magnitude and probability of reward. The reward magnitude was displayed as the number (1–5) of tokens. The reward probability was displayed as a stack of 2–8 rectangles, each representing a 10% increment. That is, probabilities ranged from 20% to 80%. Participants needed to press the left or the right button corresponding to their choice. Their choice was highlighted with a bold square for 1000 ms after their decision. This information was then replaced by a red fixation cross. The next trial started after a variable inter‐trial interval (ISI). Participants were informed that each trial was independent, that is, the outcome and/or feedback of each trial was unrelated to prior ones. They were informed that after completing each choice, the outcome of their decision (i.e., how many tokens they win) would be recorded by the computer. They were also informed that the outcome for each trial would not be presented immediately. Instead, feedback on the sum of their gains will be presented only at the end of the experiment.

***The task with one‐dimensional (1‐D) feedback***. The procedure of this task was similar as the task without immediate feedback, except that in this task, participants received feedback after each trial. The feedback was presented as a message “+ x” (i.e., win x tokens) for 1500 ms (see Figure 1b).

In the two tasks, participants finished five blocks of 28 trials. In each block, each of the 14 different reward probability‐magnitude combinations shown in Table 1 was presented twice, counterbalancing the left‐right display of the options. Before the experiment, participants were asked to practice using practice trials until they were familiar with the experimental procedure.

#### 2.1.3 Procedure

Participants completed the task without immediate feedback and the task with 1‐D feedback successively. They completed practice sessions for both tasks before the experiment, and they were told that trials in both tasks were independent, which means the outcome in previous trials would not affect the next trial.

### 2.2 Analysis

The analyses of this study were conducted using hierarchical linear modeling (HLM) (Anthony & Bryk, 1992), which allows the analysis of multiple levels simultaneously. That is, we could test for interactions between variables at different levels of analysis while accounting for their different sources of variance (Hofmann et al., 2000). The standard process for HLM is to run a series of models to test the hypotheses that relate to different levels of analysis (Hofmann et al., 2000). At the within‐subject level, or level 1 analysis, a regression equation is calculated for each participant. The mean within‐subject effects from level 1 are then used as dependent variables at the between‐subject level, or Level 2 analysis.

We first calculated the value difference in magnitude of two options (MR, Table 1) and the value difference in probability of two options (PR, Table 1) for each trial. For better analysis, we divided MR into two categories (small MR: 1 and 2; large MR: 3 and 4), and divided PR into three categories (small PR: 20%; medium PR: 40%; large PR: 60%). Here, we took MR, PR, and feedback type (without‐immediate feedback; with‐1‐D feedback) as independent variables, and choice for safer options as the dependent variable. Here, a safer option was defined as the option with a larger probability (option 1 in Table 1).

Because MR, feedback type, and choice for safer options were binary variables, they were all set to dummy variables for multilevel linear analysis (MR: small MR was set to 0 and large MR was set to 1; feedback type: without‐immediate feedback was set to 0 and with‐1‐D feedback was set to 1; choice for safer options: choosing riskier option was set to 0, choosing safer option was set to 1). PR was a three‐categorical variable, so we converted the value of each categorical into a dummy variable separately, using small PR as a reference.

### 2.3 Results

First, an empty model (***Model 0***) was run. This model examined the variance in choice before accounting for any predictors. The test of *Model 0* found that a significant proportion of variance in choice for safer options (ICC = *between‐subject‐variance/Total variance =* *μ_0j_*/[*μ_0j_* +*ε_ij_*] = 0.085 > 0.06, χ^2^ = 4.517, *p* < .001) occurred between participants. This finding indicated that multilevel modeling was appropriate. And it indicates that 8.5% of the variance in the choice for safer options was at the between‐subject level, whereas 91.5% of the variability was at the within‐subject level (see Table 2).

**TABLE 2:** HLM results for Study 1

**Models**			**Model 0**	**Model 1**
**Fixed effects (Coefficient)**	Intercept		0.819^***^	0.756^***^
	MR	Large MR		–0.090^***^
		Small MR (reference)		
	PR	Large PR		0.253^***^
		Median PR		0.213^***^
		Small PR (reference)		
	Feedback type	With‐1‐D feedback		–0.136^***^
		Without feedback (reference)		
**Random effects (Variance)**	Residual (within‐subject level error)		0.1355^***^	0.1065^***^
	Intercept (between‐subject level error)		0.0125^***^	0.0028
	MR			0.0028^***^
	PR			0.0070^***^
	Feedback type			0.0141^***^
**Information criteria**	AIC		10,970	8290
	BIC		10,985	8327

*Note*: The lower AIC (Akailke's Information Criterion) and BIC (Bozdogan's Information Criterion) indicate the better goodness of fitting model.

Abbreviation: HLM, hierarchical linear modeling.

*** *p* < .001.

Second, an unconditional model (***Model 1***) was run. This model tested the within‐subject main effects. Fixed effect coefficients were used to test these relationships. The associated variance components were used to test whether mean within‐subject effects vary significantly between participants. In this model, variables of within‐subject level (feedback type, MR, and PR) were entered as fixed and random effects.

**FIGURE 2:**
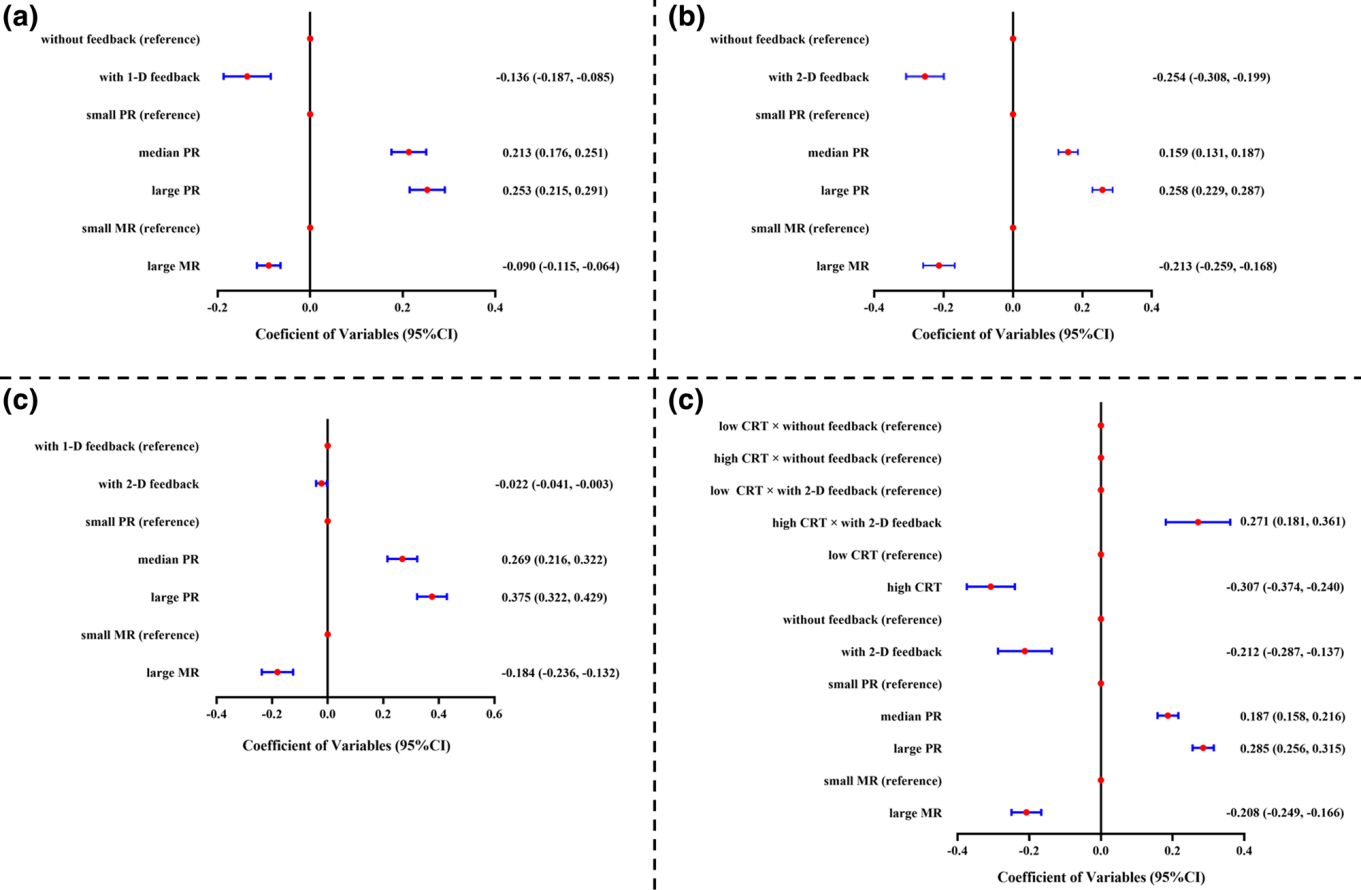
Fixed effects of final models in the four studies (a: Study 1; b: Study 2; c: Study 3; and d: Study 4). These figures intuitively showed the extent to which each variable predicted the choice for safer options. If the red dot was to the left of the zero boundary, it means that the corresponding variable’ prediction for choices of safer options is negative. Conversely, if the red dot is to the right, it means that the corresponding variable’ prediction for choices of safer options is positive.

The results of *Model 1* (see Table 2 and Figure 2a) showed that feedback type and MR were both significantly negative predictors of the choice for safer options, *t*(45) = −5.35, *p* < .001, 95% CI = [−0.187, −0.085], and *t*(45) = −7.06, *p* < .001, 95% CI = [−0.115, −0.064]. Results indicated that at the within‐subject level, the choice for safer options with‐1‐D feedback condition (*M* = 0.73, *SD* = 0.022) was lower than that in the without‐feedback condition (*M* = 0.86, *SD* = 0.022). They further showed that the choice for safer options in the large‐MR condition (*M* = 0.75, *SD* = 0.019) was lower than that in the small‐large condition (*M* = 0.84, *SD* = 0.018).

However, PR was a significantly positive predictor of the choice for safer options. Specifically, the choice for safer options increased when PR increased. The choice for safer options in median‐PR and large‐PR conditions were both significantly lower than that in the small‐PR condition, *t*(87) = 11.29, *p* < .001, 95% CI = [0.175, 0.251], and *t*(91) = 13.25, *p* < .001, 95% CI = [0.215, 0.291]. In sum, the within‐subject level main effect variables accounted for 21.4% of the within‐subject variability in choice for safer options. For *Model 1*, the AIC and BIC were all lower than those of *Model 0*, indicating that adding the variables was appropriate. Based on this, we took *Model 1* as the final model.

### 2.4 Discussion

Study 1 supported the hypothesis that in a gambling task containing explicit outcome values and corresponding probabilities, participants were less risk‐averse in the task with one‐dimensional feedback than in the task without immediate feedback. This suggested the existence of feedback immediacy effects and supported ideas of regret aversion in decision‐making (Larrick & Boles, 1995; Zeelenberg, 1999; Zeelenberg & Beattie, 1997; Zeelenberg & Pieters, 2004). In addition, we found that MR and PR contributed to the formation of participants’ decision strategy, which indicated that participants were not always fully rationale. Instead, they sometimes used heuristics (Todd, 2007) to make quick and less effortful decisions.

## 3 STUDY 2

In real life, feedback information is not always one‐dimensional, so in Study 2, we wanted to replicate and extend the main finding of Study 1 by adding additional feedback information. We specifically extended the feedback from focusing on outcome only (like in Study 1) to also accounting for the quality of the decision (“rational” or “irrational”). We assumed that participants would also show less risk aversion in the task with two‐dimensional feedback compared to that in the task without immediate feedback.

### 3.1 Method

#### 3.1.1 Participants

Forty college students (21 females; age: *M* = 19.43 years, *SD* = 1.18, range = 18–25) took part in this study. All reported having a normal or corrected‐to‐normal vision.

#### 3.1.2 Tasks and materials

***Task without immediate feedback***. This task was the same as the task without immediate feedback in Study 1 (see Figure 1a). This task consisted of five blocks of 28 trials.

***Task with two‐dimensional feedback (Task with 2‐D feedback)***. This task was like the task with 1‐D feedback in Study 1, except that in the current task, the feedback screen included two dimensions of feedback information (see Figure 1c). To avoid confusion, we used lines and colors to represent the two types of feedback. Green lines represented wins, and red lines represented losses (i.e., not win). We use wins and losses to simply represent the outcome, however; in this task, participants will always win or not win the token in each trial. The horizontal line represented a “rational” choice (i.e., choose the higher EV option), and the diagonal lines represented an “irrational” choice (i.e., choose the lower EV option). This way we created four different feedback combinations, each conveying information on a 2 (win vs. loss) × 2 (rationality vs. irrationality) grid of attributes: win & rationality; win & irrationality; loss & rationality; and loss & irrationality. In total, this task consisted of five blocks of 28 trials.

#### 3.1.3 Procedure

Participants completed the task without immediate feedback and the task with two‐dimensional feedback successively. In the task with 2‐D feedback, participants were told that “rationality” feedback means choosing such options in the long term would maximize their gains from an economic perspective, while “irrationality” feedback means that their choice was suboptimal and does not maximize long‐term gain. However, they were not provided with any information about the “rationality” or “irrationality” of specific choices during the choice phase. They needed to learn the rules in the feedback phase to help them make better decisions. Before the experiment, they practiced until they were familiar with the experimental procedure.

### 3.2 Analysis

Data analysis was the same as that in Study 1. In this study, feedback type was also set to a dummy variable (without‐immediate feedback was set to 0 and with‐2‐D feedback was set to 1). Here, we took MR, PR, and feedback type (without‐immediate feedback; with‐2‐D feedback) as independent variables and choice for safer options as the dependent variable.

### 3.3 Results

First, an empty model (***Model 0***) was run. The test of *Model 0* found that a significant proportion of variance in choice for safer options (ICC =*between‐subject‐variance/Total variance =* *μ_0j_*/[*μ_0j_* +*ε_ij_*] = 0.064 > 0.06, χ^2^ = 4.51, *p* < .001) occurred between participants. This finding indicated that multilevel modeling was appropriate. And it indicates that 6.4% of the variance in the choice for safer options was at the between‐subject level, whereas 93.6% of the variability was at the within‐subject level (see Table 3).

**TABLE 3:** HLM results for Study 2

**Models**			**Model 0**	**Model 1**
**Fixed effects (Coefficient)**	Intercept		0.715^***^	0.786^***^
	MR	Large MR		–0.213^***^
		Small MR (reference)		
	PR	Large PR		0.258^***^
		Median PR		0.159^***^
		Small PR (reference)		
	Feedback type	With‐2D feedback		–0.254^***^
		Without feedback (reference)		
**Random effects (Variance)**	Residual (within‐subject level error)		0.1908^***^	0.1365^***^
	Intercept (between‐subject level error)		0.0132^***^	0.0016
	MR			0.0108^***^
	PR			0.003^***^
	Feedback type			0.0156^***^
**Information criteria**	AIC		15,364	11,442
	BIC		15,379	11,479

*Note*: The lower AIC (Akailke's Information Criterion) and BIC (Bozdogan's Information Criterion) indicate the better goodness of fitting model.

Abbreviation: HLM, hierarchical linear modeling.

*** *p* < .001.

Second, an unconditional model (***Model 1***) was run. The results of *Model 1* (see Table 3 and Figure 2b) showed that feedback type and MR were both significantly negative predictors of the choice for safer options, *t*(45) = −9.43, *p* < .001, and *t*(45) = −9.39, *p* < .001. Respectively, it indicated that within‐subject level, the choice for safer options in with‐2‐D feedback condition (*M* = 0.56, *SD* = 0.023) was less than that in the without‐feedback condition (*M* = 0.82, *SD* = 0.023), and the choice for safer options in large‐MR condition (*M* = 0.58, *SD* = 0.022) was less than that in small‐large condition (*M* = 0.79, *SD* = 0.022). However, PR was a significantly positive predictor of the choice for safer options. Specifically, the choice for safer options increased with PR increased. The choice for safer options in median‐PR and large‐PR conditions were both significant less than that in small‐PR condition, *t*(85) = 11.21, *p* < .001, and *t*(93) = 17.84, *p* < .001. These findings were consistent with those in Study 1. In sum, the with‐subject level’ main effect variables accounted for 28.5% of the within‐subject variability in choice for safer options. For *Model 1*, the AIC and BIC were all lower than *Model 0*, indicating that adding the variables was appropriate. Based on this, we took *Model 1* as the final model.

### 3.4 Discussion

The present study provided stronger support to **H1** by replicating the effect of feedback immediacy on risk‐taking, which again indicated the existence of regret aversion effect (Larrick & Boles, 1995; Zeelenberg, 1999; Zeelenberg & Beattie, 1997; Zeelenberg & Pieters, 2004). Consistent with Study 1, we also found the effects of MR and PR on risk‐taking, which provided further support to the view that participants used simple and fast heuristics (Todd, 2007) to reach quick decisions.

## 4 STUDY 3

The aim of Study 3 was to examine **H2** by comparing the effect of one‐dimensional feedback and two‐dimensional feedback on risk‐taking. We posited that participants would show a difference in choices between the two tasks.

### 4.1 Method

#### 4.1.1 Participants

Forty‐two college students participated in this study (25 females; age: *M* = 19.58 years, *SD* = 1.47, ranging = 18–24). All reported having a normal or corrected‐to‐normal vision.

#### 4.1.2 Tasks and materials

***Task with one‐dimensional feedback (task with 1‐D feedback)***. This task was the same as the task with 1‐D feedback in Study 1 (see Figure 1b). This task consisted of five blocks of 28 trials.

***Task with two‐dimensional feedback (Task with 2‐D feedback)***. This task was the same as the task in Study 2 (see Figure 1c). This task consisted of five blocks of 28 trials.

#### 4.1.3 Procedure

Participants completed the task without 1‐D feedback (same as in Study 1) and the task with 2‐D feedback (same as in Study 2) successively. Before the experiment, they practiced until they were familiar with the experimental procedure.

### 4.2 Analysis

The behavior analysis was the same as that in Study 1. In this study, feedback type was also set to a dummy variable (“with 1‐D feedback” was set to 0, and “with 2‐D feedback” was set to 1). Here, we took MR, PR, and feedback type (without‐1‐D feedback; with‐2‐D feedback) as independent variables and choice for safer options as the dependent variable.

### 4.3 Results

First, an empty model (***Model 0***) was run. The test of *Model 0* found that a significant proportion of variance in choice for safer options (ICC = *between‐subject‐variance/Total variance =* *μ_0j_*/[*μ_0j_* +*ε_ij_*] = 0.123 > 0.06, χ^2^ = 4.416, *p* < .001) occurred between participants. This finding indicated that multilevel modeling was appropriate (Wen, 2009). And it indicates that 12.3% of the variance in the choice for safer options was at the between‐subject level, whereas 87.7% of the variability was at the within‐subject level (see Table 4).

**TABLE 4:** HLM results for Study 3

**Models**			**Model 0**	**Model 1**
**Fixed effects (Coefficient)**	Intercept		0.682^**^	0.536^**^
	MR	Large MR		–0.184^**^
		Small MR (reference)		
	PR	Large PR		0.375^**^
		Median PR		0.269^**^
		Small PR (reference)		
	Feedback type	With‐2‐D feedback		–0.022^*^
		With‐1‐D feedback (reference)		
**Random effects (Variance)**	Residual (within‐subject level error)		0.191^**^	0.1484^**^
	Intercept (between‐subject level error)		0.0268^**^	0.0184^*^
	MR			0.0128^**^
	PR			0.0135^**^
	Feedback type			0.0008^*^
**Information criteria**	AIC		14,056	11,458
	BIC		14,070	11,495

*Note*: The lower AIC (Akailke's Information Criterion) and BIC (Bozdogan's Information Criterion) indicate the better goodness of fitting model.

Abbreviation: HLM, hierarchical linear modeling.

*p < .05; **p < .01.

Second, an unconditional model (***Model 1***) was run. The results of *Model 1* (see Table 4 and Figure 2c) showed that feedback type and MR were both significantly negative predictors of the choice for safer options, *t*(41) = −2.32, *p* = .025, and *t*(41) = −7.12, *p* < .001. Respectively, it indicated that within‐subject level, the choice for safer options in 2‐D feedback condition (*M* = 0.64, *SD* = 0.027) was less than that in the 1‐D feedback condition (*M* = 0.70, *SD* = 0.027), and the choice for safer options in large‐MR condition (*M* = 0.56, *SD* = 0.03) was less than that in the small‐large condition (*M* = 0.74, *SD* = 0.03). However, PR was a significantly positive predictor of the choice for safer options. Specifically, the choice for safer options increased with PR increased. The choice for safer options in median‐PR and large‐PR conditions were both significantly less than that in the small‐PR condition, *t*(80) = 10.01, *p* < .001, and *t*(82) = 13.87, *p* < .001. In sum, the with‐subject level’ main effect variables accounted for 22.3% of the within‐subject variability in choice for safer options. For *Model 1*, the AIC and BIC were all lower than *Model 0*, indicating that adding the variables was appropriate. Based on this, we took *Model 1* as the final model.

### 4.4 Discussion

The results of this study supported H2. Specifically, participants showed less risk aversion in the task with two‐dimensional feedback than in the task with one‐dimensional feedback, but their risk preferences were still not fully rational. This supports the regret effect idea and the “more important dimension” hypothesis. It suggests that participants’ choices might be influenced by the two dimensions of feedback, and that participants might give priority to one of the two dimensions, namely, the outcome of decision under uncertainty conditions.

## 5 STUDY 4

The results of Study 3 have shown that the effect of feedback immediacy was indeed enhanced with the expansion of immediate feedback. Given the assumption that CRT‐score groups may differ in their motivations to attend to feedback information, we examined the hypothesis that the choices would be moderated by participants’ cognitive reflection ability. To capture participants’ cognitive reflection ability, we employed the CRT by Frederick (2005). This test includes three questions with possible score from 0 to 3. Following the procedure in Frederick (2005), we divided participants into two groups based on their CRT performance. One was the “Low” CRT group, which were those participants who correctly answered one question or less on the CRT (scores = 0 or 1), the other was the “High” CRT group, which included participants who correctly answered 2–3 questions (scores = 2 or 3). We considered the “Low” CRT group as a subset of cognitive misers, whereas the “High” CRT group was conceived to be part of more reflective decision‐makers. Frederick (2005) showed that CRT performance significantly correlates with risk preference, that is, more reflective participants are, on average, less risk‐averse and more patient.

### 5.1 Method

#### 5.1.1 Participants

Fifty‐three college students (27 participants with high CRT scores, 26 participants with low CRT scores; their age: *M* = 21.14 years, ranging = 19–25) took part in the experiment. All participants reported having a normal or corrected‐to‐normal vision.

#### 5.1.2 Tasks and materials

***Cognitive reflection test***. Participants were asked about the following three questions, and they were instructed to answer these questions quickly. (1) A bat and a ball cost $1.10 in total. The bat costs a dollar more than the ball. How much does the ball cost? ____ cents. (2) If it takes 5 machines 5 min to make 5 widgets, how long would it take 100 machines to make 100 widgets? ____ min. (3) In a lake, there is a patch of lily pads. Every day, the patch doubles in size. If it takes 48 days for the patch to cover the entire lake, how long would it take for the patch to cover half of the lake? ____ days.

***Task without immediate feedback***. This task was the same as the task without immediate feedback in Study 1 (see Figure 1a).

***Task with two‐dimensional feedback (Task with 2‐D feedback)***. This task was the same as the task in Study 2 (see Figure 1c).

#### 5.1.3 Procedure

Before the experiment, we divided the participants into “Low” and “High” CRT groups based on their CRT scores. Next, all participants completed the task without immediate feedback and the task with 2‐D feedback successively. Before the experiment, participants were asked to practice until they were familiar with the experimental procedure.

### 5.2 Analysis

Data analysis was almost the same as that in Study 1. In this study, feedback type was also set to a dummy variable (without‐immediate feedback was set to 0 and with‐2‐D feedback was set to 1), and the between‐subject variable of CRT was set to dummy variable (low CRT was set to 0 and high CRT was set to 1). Here, we took MR, PR, feedback type (without‐immediate feedback; with‐1‐D feedback), and CRT (high; low) and took the choice for safer options as the dependent variable.

### 5.3 Results

First, an empty model (***Model 0***) was run. The test of *Model 0* found that a significant proportion of variance in choice for safer options (ICC = *between‐subject‐variance/Total variance =* *μ_0j_*/[*μ_0j_* +*ε_ij_*] = 0.0655 > 0.06, χ^2^ = 4.756, *p* < .001) occurred between participants. This finding indicated that multilevel modeling was appropriate. And it indicates that 6.55% of the variance in the choice for safer options was at the between‐subject level, whereas 93.45% of the variability was at the within‐subject level (see Table 5).

**TABLE 5:** HLM results for Study 4

**Models**			**Model 0**	**Model 1**	**Model 2**
**Fixed effects (Coefficient)**	Intercept		0.646^***^	0.604^***^	0.758^***^
	MR	Large MR		–0.208^***^	–0.208^***^
		Small MR (reference)			
	PR	Large PR		0.285^***^	0.285^***^
		Median PR		0.187^***^	0.187^***^
		Small PR (reference)			
	Feedback type	With‐2‐D feedback		–0.076^***^	–0.212^***^
Without feedback (reference)					
	CRT	High CRT			–0.307^***^
		Low CRT (reference)			
	Feedback type × CRT				0.271^***^
**Random effects (Variance)**	Residual (within‐subject level error)		0.2138^***^	0.1702^***^	0.1694^***^
	Intercept (between‐subject level error)		0.0149^***^	0.0003	0.0003
	MR			0.0103^***^	0.0097^***^
	PR			0.0037^***^	0.0036^***^
	Feedback type			0.0247^***^	0.0217^***^
**Information criteria**	AIC		19,390	16,431	16,363
	BIC		19,406	16,469	16,408

*Note*: The lower AIC (Akailke's Information Criterion) and BIC (Bozdogan's Information Criterion) indicate the better goodness of fitting model.

Abbreviation: HLM, hierarchical linear modeling.

^***^ *p* < .001.

Second, an unconditional model (***Model 1***) was run. The results of *Model 1* (see Table 5) showed that feedback type and MR were both significantly negative predictors of the choice for safer options, *t*(72) = −2.40, *p* = .019, 95% CI = −0.14∼−0.013, and *t*(54) = −9.72, *p* < .001, 95% CI = −0.251∼−0.165. Respectively, it indicated that within‐subject level, the choice for safer options in with‐2‐D feedback condition (*M* = 0.581, *SD* = 0.025) was less than that in without‐feedback condition (*M* = 0.658, *SD* = 0.025), and the choice for safer options in large‐MR condition (*M* = 0.516, *SD* = 0.022) was less than that in small‐large condition (*M* = 0.724, *SD* = 0.022). However, PR was a significantly positive predictor of the choice for safer options. Specifically, the choice for safer options increased with PR increased. The choice for safer options in median‐PR and large‐PR conditions were both significantly less than that in small‐PR condition, *t*(93) = 12.78, *p* < .001, 95% CI = 0.158−0.216, and *t*(102) = 19.06, *p* < .001, 95% CI = 0.256−0.315. In sum, the with‐subject level’ main effect variables accounted for 20.4% of the within‐subject variability in choice for safer options.

A conditional model was run. Based on *Model 1*, between‐subject level’ variable of CRT was computed to *Model 2* as a fixed effect. We tested whether the effect of feedback was moderated by CRT. The results of ***Model 2*** (see Table 5 and Figure 2d) showed that feedback type, MR, and CRT were all significantly negative predictors of the choice for safer options, *t*(99) = −5.63, *p* < .001, 95% CI = −0.287∼−0.137; *t*(59) = −9.97, *p* < .001, 95% CI = −0.249∼−0.166; *t*(393) = −8.98, *p* < .001, 95% CI = −0.374∼−0.24. And PR was a significantly positive predictor of the choice for safer options. Furthermore, the interaction term of feedback type and CRT was found, *t*(218) = 5.94, *p* < .001, 95% CI = 0.181−0.361. Follow‐up simple analysis showed that for participants with low CRT, feedback type positively predicted the choice for safer options (*β* = 0.799, *p* < .001); however, for participants with high CRT, feedback type did not link to the choice for safer options (*β* = 0.0076, *p* = .87). Participants with low CRT showed a significant difference in the choice for safer options between the task without immediate feedback (*M* = 0.599, *SD* = 0.029) and the task with 2‐D feedback (*M* = 0.812, *SD* = 0.029), but the participants with high CRT did not show any significant difference between the task without immediate feedback (*M* = 0.504, *SD* = 0.029) and the task with 2‐D feedback (*M* = 0.563, *SD* = 0.029). In sum, the between‐subject level’ variable of CRT explained 10.93% of the variability in the feedback type effect.

Overall, compared with other models, *Model 2* had the lowest BIC and AIC, indicating that the goodness of *Model 2* was the best (see Table 5). Based on this, we took *Model 2* as the final model.

### 5.4 Discussion

The results of Study 4 supported **H3**. They showed that the effect of feedback immediacy was moderated by participants’ cognitive reflection. Feedback immediacy influenced risk‐taking for participants low in CRT, but not in participants high in CRT. This supported ideas regarding the role of cognitive reflection ability in risky decision‐making, and again supported the existence of feedback immediacy effect.

## 6 GENERAL DISCUSSION

In this paper, we investigated nuanced insights related to the effect of feedback immediacy on risk‐taking. We found that participants showed less risk aversion in tasks with immediate feedback (one‐dimension feedback and two‐dimension feedback) compared to when they engaged in decision tasks without‐immediate feedback. This provided support to the regret aversion effect (Larrick & Boles, 1995; Zeelenberg, 1999; Zeelenberg & Beattie, 1997; Zeelenberg & Pieters, 2004). In addition, the reduction in risk‐averse behaviors was greater in the task with two‐dimensional feedback than that in the task with one‐dimensional feedback. Despite this further reduction, participants’ risk preference in the task with two‐dimensional feedback did not reach fully rationality. This finding supports the “more important dimension” hypothesis (Kray & Gonzalez, 1999; Slovic, 1975). Lastly, we found that immediate feedback effects on risk‐taking were moderated by one's cognitive reflection ability, as measured by CRT. We discuss these findings below.

### 6.1 The effect of feedback in decision‐making under risk

In this paper, we have deepened the understanding of the effect of immediate feedback on risk‐taking. In Study 1, we observed that participants were less risk‐averse in the task with immediate feedback. The same results were obtained in Study 2. This supported the regret aversion effect. That is, the manipulation of feedback information might have induced participants’ regret aversion. As mentioned in the introduction, regret is a functional emotion that can help participants learn from prior mistakes (Zeelenberg, 1999), and people make choices that aim at minimizing regret rather than minimizing risk (Zeelenberg et al., 1996). In our study, feedback information enabled participants to obtain information about the quality of prior decisions, and to see the difference between the actual outcome and the expected outcome. This probably drove them to adjust their choice selection strategy. Participants showed a similar selection strategy in decision‐making across the four studies, which might indicate a general pattern when participants make decisions in money‐related gambling tasks. Specifically, we found that the larger the magnitude difference between the two options was, the less risk‐averse the participants were and the larger the probability difference between the two options was, the more risk‐averse the participants were. This might indicate that when making decisions in money‐related gambling tasks, participants usually use simple and fast heuristics rather than following the rules of full, cold “rationality.” Our findings show that participants’ choices were mostly simply according to the magnitude difference and the probability difference between the two options, especially in gambling tasks without feedback.

In Study 3, we found that the effect of feedback was enhanced with the expansion of feedback dimensionally. That is, participants showed less risk aversion in the task with two‐dimensional feedback than in the task with one‐dimensional feedback. Nevertheless, participants’ choices in the task with two‐dimensional feedback did not reach to fully rationality. In the task with two‐dimensional feedback, one dimension of feedback was the outcome of the decision (win/loss), and the other was the quality of decision (rationality/irrationality). Given that the feedback on quality provided the information that could induce participants to be more rational, it might be a reason why participants showed less risk aversion and even trended toward fully rationality when receiving such feedback.

The “more important dimension” hypothesis (Kray & Gonzalez, 1999; Slovic, 1975) might explain why participants showed less risk aversion but not fully rational in the task with two‐dimension feedback. Specifically, in this task, although participants obtained two feedback dimensions, they likely tried to conserve resources and focused mainly on one feedback dimensions, namely, the outcome of decision (win/loss). Therefore, they spent less cognitive resources on the other feedback dimension, that is, the quality of decision (rationality/irrationality). That is, the outcome of decision (win/loss) was more important feedback dimension for participants.

In addition to the effect of feedback on risk‐taking, we also found that this effect was moderated by participants’ cognitive reflection ability. In Study 4, participants with low cognitive reflection ability showed a significant difference in choices between the task without immediate feedback and the task with two‐dimensional feedback. Specifically, they showed less risk aversion in the task with two‐dimensional feedback, but no effect of feedback immediacy was found in the participants high in cognitive reflection ability. In other words, the manipulation of feedback was successful in participants with low rather than high cognitive reflection ability. These findings not only validated the existence of immediate feedback effects, but also supported previous studies on the relationship between risk aversion and cognitive reflection ability (Andersson et al., 2016; Benjamin et al., 2013; Carlos et al., 2016; Donkers et al., 2001; Frederick, 2005; Gill, 2015).

These findings suggest that participants with low cognitive reflection ability adjusted their selection strategy in the task with two‐dimensional feedback and thus their choices were closer to fully rationality. This indicated that they might be influenced by the feedback on the outcome of their decision (i.e., win/loss) and learned the rules of normative choices from the feedback on the quality of decision (i.e., rationality/irrationality). However, they showed a selection strategy to be risk‐averse in the task without immediate feedback. For participants with high cognitive reflection ability, the risk preference was consistent between the two tasks. They were both relatively less risk‐averse and even closer to risk‐neutral in the two tasks.

In the context of the dual‐system theories (Evans & Stanovich, 2013; Kahneman & Frederick, 2002; Lilleholt, 2019; Loewenstein & O'Donoghue, 2004), people's latent risk preference is partly driven by the emotional System 1, and high cognitive reflection ability entails greater control of decisions by the deliberative System 2. Participants with higher reflection ability have more cognitive capacity to consciously reflect on their choices, so their choices are usually more normative (Frederick, 2005; Lilleholt, 2019; Oechssler et al., 2009).

In our study, the change in risk preference for participants with low cognitive ability from risk aversion to near but not reach rational required feedback intervention. This finding was consistent with that in Study 3 and proved again the regret effect and the “more important dimension” hypothesis. Nevertheless, previous studies have not found that participants with low CRT behave more rational in gambling tasks, even in the tasks with feedback (Andersson et al., 2016; Benjamin et al., 2013; Carlos et al., 2016; Donkers et al., 2001; Frederick, 2005; Gill, 2015). An explanation for this might be the difference in task feedback information. Specifically, in most previous studies, feedback in gambling tasks only contained the outcome of decision (e.g., win/loss). In contrast, in our study, feedback in the gambling task contained not only the outcome of decision but also the quality of decision (i.e., rationality/irrationality). Overall, we concluded that the quality of decision, to a certain degree, contributed to the change of choices in participants with low cognitive ability between the task without immediate feedback and the task with two‐dimensional feedback, in particular, a shift from risk aversion to close to rationality.

### 6.2 Limitations and future directions

Several limitations of our study are noteworthy. First, our sample was restricted to students who reside in one country. The generalizability of our results should be established through replication with other populations. Second, our task represented only one type of risk‐taking. Generalizability to other situations, for example, when more dimensions of feedback are provided (e.g., about what others have done), should be established in future research by employing different risk‐taking paradigms. Lastly, although we use regret aversion and cognitive load minimization to explain our results, we did not directly measure them. Future research should examine such mediational mechanisms and extend our models.

### PEER REVIEW

The peer review history for this article is available at https://publons.com/publon/10.1002/brb3.2957.

## Data Availability

The data that support the findings of this study are available from the corresponding author upon reasonable request.

## References

[brb32957-bib-0025] Andersson, O. , Holm, H. J. , Tyran, J.‐R. , & Wengstrom, E. (2016). Risk aversion relates to cognitive ability: Preference or noise? Journal of the European Economic Association, 14(5), 1129–1154.

[brb32957-bib-0001] Anthony, S. , & Bryk, S. W. R. (1992). Hierarchical linear models: Applications and data analysis methods. Publications of the American Statistical Association.

[brb32957-bib-0002] Benjamin, D. J. , Brown, S. A. , & Shapiro, J. M. (2013). Who is ‘behavioral’? Cognitive ability and anomalous preferences. Levine's Working Paper Archive, 11(6), 1231–1255. 10.1111/jeea.12055 PMC6289538 30546272

[brb32957-bib-0003] Berndsen, M. , Pligt, J. V. D. , Doosje, B. , & Manstead, A. (2004). Guilt and regret: The determining role of interpersonal and intrapersonal harm. Cognition & Emotion, 18(1), 55–70.

[brb32957-bib-0004] Brand, M. (2008). Does the feedback from previous trials influence current decisions? A study on the role of feedback processing in making decisions under explicit risk conditions. Journal of Neuropsychology, 2(2), 431–443. 19824172 10.1348/174866407x220607

[brb32957-bib-0005] Carlos, C. , Iturbe‐Ormaetxe, I. , Mata‐Pérez, E. , Ponti, G. , Sartarelli, M. , Yu, H. , & Zhukova, V. (2016). Cognitive (ir)reflection: New experimental evidence. Journal of Behavioral & Experimental Economics, 64, 81–93.

[brb32957-bib-0006] Conway, A. R. A. , Kane, M. J. , & Engle, R. W. (2003). Working memory capacity and its relation to general intelligence. Trends in Cognitive Sciences, 7(12), 547–552. 14643371 10.1016/j.tics.2003.10.005

[brb32957-bib-0008] Dijk, W. W. V. , & Zeelenberg, M. (2002). Investigating the appraisal patterns of regret and disappointment. Motivation & Emotion, 26(4), 321–331.

[brb32957-bib-0009] Donkers, B. , Melenberg, B. , & Soest, A. V. (2001). Estimating risk attitudes using lotteries: A large sample approach. Journal of Risk & Uncertainty, 22(2), 165–195.

[brb32957-bib-0010] Ernst, M. , & Paulus, M. P. (2005). Neurobiology of decision making: A selective review from a neurocognitive and clinical perspective. Biological Psychiatry, 58(8), 597–604. 16095567 10.1016/j.biopsych.2005.06.004

[brb32957-bib-0011] Evans, J. S. B. T. , & Stanovich, K. E. (2013). Dual‐process theories of higher cognition: Advancing the debate. Perspectives on Psychological Science, 8(3), 223–241. 26172965 10.1177/1745691612460685

[brb32957-bib-0012] Frankish, K. (2010). Dual‐process and dual‐system theories of reasoning. Philosophy Compass, 5(10), 914–926.

[brb32957-bib-0013] Frederick, S. (2005). Cognitive reflection and decision making. Journal of Economic Perspectives, 19(4), 25–42.

[brb32957-bib-0014] Gill, D. & Prowse, V. (2016). Cognitive ability, character skills, and learning to play equilibrium: A level‐k analysis. Journal of Political Economy, 124(6), 1619–1676.

[brb32957-bib-0015] Hofmann, S. A. , Griffin, M. , & Gavin, M. B. (2000). The application of hierarchical linear modeling to organizational research. In K. J. Klein , & W. J. Kozlowski (Eds.), Multilevel theory, research, and methods in organizations: Foundations, extensions, and new directions (pp. 467–511). Jossey‐Bass/Wiley.

[brb32957-bib-0016] Ilgen, D. R. , & Moore, C. F. (1987). Types and choices of performance feedback. Journal of Applied Psychology, 72(3), 401–406.

[brb32957-bib-0017] Janis, I. I. , & Mann, L. (1977). Decisional problems: Decision making. A psychological analysis of conflict, choice, and commitment. Science, 197, 1355–1356.17747001

[brb32957-bib-0018] Kahneman, D. (2011). Thinking, fast and slow. New York: Farrar, Straus and Giroux.

[brb32957-bib-0019] Kahneman, D. , & Frederick, S. (2002). Representativeness revisited: Attribute substitution in intuitive judgement. In T. Gilovich , D. Griffin , & D. Kahneman (Eds.). Heuristics and biases: The psychology of intuitive judgement (pp. 49–81). Cambridge: Cambridge University Press.

[brb32957-bib-0020] Kray, L. , & Gonzalez, R. (1999). Differential weighting in choice versus advice: I'll do this, you do that. Journal of Behavioral Decision Making, 12(3), 207–218.

[brb32957-bib-0021] Larrick, R. P. , & Boles, T. L. (1995). Avoiding regret in decisions with feedback: A negotiation example. Organizational Behavior & Human Decision Processes, 63(1), 87–97.

[brb32957-bib-0022] Lilleholt, L. (2019). Cognitive ability and risk aversion: A systematic review and meta analysis. Judgment and Decision Making, 14(3), 234–279.

[brb32957-bib-0023] Loewenstein, G. , & O'Donoghue, T. (2004). Animal spirits: Affective and deliberative processes in economic behavior. Working Papers.

[brb32957-bib-0024] Oechssler, J. R. , Schmitz, A. , & Patrick, P. W. (2009). Cognitive abilities and behavioral biases. Journal of Economic Behavior & Organization, 72(1), 147–152.

[brb32957-bib-0026] Pleskac, T. J. (2008). Decision making and learning while taking sequential risks. Journal of Experimental Psychology Learning Memory and Cognition, 34(1), 167–185. 18194061 10.1037/0278-7393.34.1.167

[brb32957-bib-0027] Rabin, M. , & Thaler, R. H. (2001). Abnormalies: Risk aversion. Journal of Economic Perspectives, 15(1), 219–232.

[brb32957-bib-0028] Sharp, M. E. , Jayalakshmi, V. , Lanyon, L. J. , Barton, J. J. S. , & Kevin, P. (2012). Sensitivity and bias in decision‐making under risk: Evaluating the perception of reward, its probability and value. PLoS ONE, 7(4), e33460. 22493669 10.1371/journal.pone.0033460 PMC3320893

[brb32957-bib-0029] Simon, H. A. (1955). A behavioral model of rational choice. Quarterly Journal of Economics, 65(1), 99–118.

[brb32957-bib-0030] Slovic, P. (1975). Choice between equally valued alternatives. Journal of Experimental Psychology Human Perception & Performance, 1(3), 280–287.

[brb32957-bib-0031] Süß, H.‐M. , Oberauer, K. , Wittmann, W. W. , Wilhelm, O. , & Schulze, R. (2002). Working‐memory capacity explains reasoning ability—And a little bit more. Intelligence, 30(3), 261–288.

[brb32957-bib-0032] Tochkov, K. (2009). The effects of anticipated regret on risk preferences of social and problem gamblers. Judgment and Decision Making, 4(3), 227.

[brb32957-bib-0033] Todd, P. M. (2007). How much information do we need? European Journal of Operational Research, 177(3), 1317–1332.

[brb32957-bib-0034] Tversky, A. , & Kahneman, D. (1974). Judgment under uncertainty: Heuristics and biases. Science, 185(4157), 1124–1131. 17835457 10.1126/science.185.4157.1124

[brb32957-bib-0035] Zeelenberg, C. M. (2002). Regret in decision making. Current Directions in Psychological Science, 11(6), 212–216.

[brb32957-bib-0036] Zeelenberg, M. (1999). The use of crying over spilled milk: A note on the rationality and functionality of regret. Philosophical Psychology, 12(3), 325–340.

[brb32957-bib-0037] Zeelenberg, M. , & Beattie, J. (1997). Consequences of regret aversion 2: Additional evidence for effects of feedback on decision making. Organizational Behavior and Human Decision Processes, 72(1), 63–78.

[brb32957-bib-0038] Zeelenberg, M. , Beattie, J. , Van der Pligt, J. , & De Vries, N. K. (1996). Consequences of regret aversion: Effects of expected feedback on risky decision making. Organizational Behavior and Human Decision Processes, 65(2), 148–158.

[brb32957-bib-0039] Zeelenberg, M. , & Pieters, R. (2004). Consequences of regret aversion in real life: The case of the Dutch postcode lottery. Organizational Behavior & Human Decision Processes, 93(2), 155–168.

